# Minutes of the Mississippi Valley Association of Dental Surgeons

**Published:** 1886-04

**Authors:** 


Adjourned.

AFTERNOON SESSION.

This session was devoted to the exhibition of appliances by Drs. McKellops and W. S. How, mention of which will be found in "Salmagundi." After the payment of bills, appropriations, etc., the society elected its officers for the ensuing year and adjourned to meet the first Wednesday of March, 1887.

### PROCEEDINGS.

### Minutes of the Mississippi Valley Association of Dental Surgeons.

FIRST DAY—MORNING SESSION, MARCH 3, 1886.

The annual meeting of the Mississippi Valley Association of Dental Surgeons was held in the hall of the Ohio Dental College, on the 3d of March, 1886, with the President, Dr. A. F. Emmenger, occupying the chair, and Dr. A. G. Rose, acting as Secretary.

The President called the association to order at 10:30 A. M., and requested Dr. W. S. Horr to open the meeting with prayer.

The Secretary read the minutes of the previous annual meeting, and there being no objection, were adopted as read.

Dr. H. L. Moore for the Executive Committee, reported the following programme for discussion:

The committees on "Ethics," "Publication" and "Membership" had no special report to make, and were continued during session.

Dr. A. G. Rose, of Cincinnati, read a paper upon the treatment of pulpless teeth, and was followed by Dr. G. W. Smith, of Cincinnati, with a paper upon "Hydrogen Per Oxide," as a remedy for the treatment of pulpless teeth.

Dr. H. A. Smith, of Cincinnati, read a paper upon "Antiseptics or Germicides" in connection with the treatment of pulpless teeth.

Drs. H. A. Smith and J. L. Cassidy discussed the subject for a few minutes until time of adjournment arrived.

On motion the following hours for sessions were adopted: From 9 A. M. to 12 M., 2 to 6 P. M.

Adjourned.

### FIRST DAY—AFTERNOON SESSION.

Dr. J. M. Jay read a paper upon the first subject of the programme, "Treatment of Pulpless Teeth."

Topic discussed by Drs. H. A. Smith, H. J. McKellops, F. A. Sage, M. H. Fletcher, G. W. Smith, A. O. Rawls.

On motion the first subject was passed and the sixth subject taken up, "Amalgam Fillings from the Standpoint of To-Day." Drs. C. M. Wright, of Cincinnati, and George Watt, of Xenia, Ohio, entertained the society with papers upon that subject.

Dr. J. Taft moved that all papers read before this association be placed in the hands of Secretary, and given to Publication Committee for the use of journals.

Sixth subject discussed by Drs. Sage, McKellops, Osmond, Leslie, Geo. Watt, of Xenia.

### SECOND DAY—MORNING SESSION.

Meeting called to order with President, Dr. A. F. Emminger in the chair, and the Secretary at his post.

Minutes of previous meeting read and adopted.

The sixth subject was passed, upon a motion being made to that effect.

Dr. M. H. Fletcher moved that the fifth subject be taken up for hearing.

Dr. J. W. Jay reported a case of death by lodgment of a partial gold denture in the brochical tubes, and read a paper upon the subject.

Dr. H. J. McKellops, of St. Louis, reported a similar case of death by lodgment of a plate, for the removal of which an operation was performed by Sir Wm. McCormick, of London.

Dr. A. Berry protested against the publication of these cases in public prints; thought the public and our patients might become alarmed. Reported a case of plate lodging in the "Esophagus," but relief was obtained by pushing the plate into the stomach, and it was eventually passed.

Dr. Berry mentioned the case of Dr. M. B. Wright, of Cincinnati, whose death was hastened by a plate lodging in esophagus.

Dr. W. C. Collins, of College Hill, Ohio, exhibited a plate which he had accidentally swallowed, and which remained in his stomach for three days, and was finally passed. Subject was discussed by Drs. Osmond, H. L. Moore, W. C. Collins, Hall, Smith, G. W. Smith, G. W. Keely.

Subject was passed upon motion.

Dr. J. Taft read a paper written by Dr. J. E. Low at the request of the Executive Committee, upon the third subject of the programme, "Crown and Bridge Work," being an interesting description of the "Low" method of constructing "Bridge Work and Crowning Roots." Models and appliances were shown, illustrating methods of work.

Dr. H. L. Moore moved that a vote of thanks be sent to Dr. Low for his interesting paper and models.

Carried.

Topic discussed by Drs. G. W. Keely, McKellopps, Rehwinkel, of Chillicothe; Van Antwerp and Berry.

Dr. C. M. Wright seconded by Dr. G. W. Kelly, moved that the question of sending a vote of thanks to Dr. J. E. Low, of Chicago, be reconsidered.

After much discussion by Drs. Taft, James, Fletcher, McKellops, Jay, Rehwinkel, the question of reconsideration was put and carried.

Dr. Good suggested that all the gentlemen who contributed papers to the association be thanked.

Rehwinkel moved that Dr. Geo. Watt be made an honorary member of this association for life.

Carried unanimously.

Dr. Watt thanked the society for the honor conferred.

The subject of BridgeWork was resumed. Dr. Ullery, of Rising Sun, Ind., being called for spoke at length about Bridge Work, as he made it over forty years ago, describing his methods of soldering porcelain teeth to a strap, and attaching to adjoining teeth or to teeth close by.

The Treasurer, Dr. F. A. Hunter, made his report. Balance, $45.75, and asked for a committee to audit his accounts.

Dr. H. A. Smith moved that 3 o'clock P. M., be set aside for the exhibition of appliances.

Carried.

Dr. C. M. Wright, of Cincinnati, seconded by Dr. G. W. Keely, Oxford, O., offered the following resolution:

"*Resolved*, That this association tenders a vote of thanks to Drs. Watt and Low, not an being active member of this society, for their kind compliance with the request of the Executive Committee, in furnishing interesting papers for this meeting of this association.

Carried unanimously.

Dr. E. G. Betty offered the following resolution:

"*Resolved*, That the sum of $25 be set apart to purchase a suitable "Gold Medal" to be awarded as a prize to any member of this association, who shall prepare for and read at the forty-third annual meeting of this body, the best essay upon any subject pertaining to the profession of dentistry, the subject to be at the discretion of the essayist. All essays written to be reported to the Executive Committee three months in advance of meeting.

Carried.

SECOND DAY—AFTERNOON SESSION.

Drs. Van Antwerp and J. R Callahan were appointed to audit the accounts of the Treasurer.

Upon motion Dr. W. S. How, of the S. S. White Company, of Philadelphia, was given one half hour to exhibit appliances for making bridge and crown work, exhibiting methods and models.

Dr. McKellops entertained the society by exhibition of appliances for crown and bridge work. Called the attention of the society to Haswell's manual, Miller's matrix, and mandrels for mounting disks, and showed some excavators of peculiar construction.

Dr. Rehwinkel, of Chillicothe, spoke at some length upon the Herbst method of filling teeth with gold.

The Vice President, Dr. E. G. Betty, here took the chair, and the Secretary reported bills amounting to $12.50 for printing, etc., which were ordered paid.

Dr. H. A. Smith moved that an appropriation of $5 be made the janitor.

Carried.

Dr. J. S. Cassidy moved that an appropriation of $15 be made the Secretary for his services.

Carried.

Auditing Committee report the Treasurer's accounts all O. K.

Wm. Van Antwerp,
J. R. Callahan.

Report accepted.

Dr. W. C. Duckwall, of Hillsboro, Ohio, was elected to membership by the Secretary's ballot.

The society proceeded to the election of officers by ballot, which resulted in the choice of the following:

President—E. G. Betty.

First Vice President—H. L. Moore.

Second Vice President—J R. Callahan.

Corresponding Secretary—A. G. Rose.

Recording Secretary—M. H. Fletcher.

Treasurer—F. A. Hunter.

A vote of thanks to retiring officers was proposed and carried.

The President appointed the following committees:

Executive Committee--F. A. Hunter, C. M. Wright. N. S. Hoff.

Publication—J. Taft, Chas. Welch, A. O. Rawls.

Membership—G. W. Smith, H. A. Beamer, R. J. Corson.

Ethics—J. W. Jay, F. C. Runyon, J. F. Dennis.

Adjourned to meet first Wednesday in March, 1887.

A. G. Rose,
Recording Secretary.

